# Low‐Energy Delicate Pulsed Light Therapy for Sensitive Skin: A Retrospective Study

**DOI:** 10.1111/jocd.16781

**Published:** 2025-01-08

**Authors:** Yujie Fu, Yue Quan, Wanxing Zhao, Zhaoyang Liu, Jiao Peng, Xinyue Pang, Boyu Zhao, Lisha Tan, Quan Zhou, Lili Shao, Huiping Wang, Shuping Hou

**Affiliations:** ^1^ Department of Dermatovenereology Tianjin Medical University General Hospital/Tianjin Institute of Sexually Transmitted Disease Tianjin China

**Keywords:** delicate pulsed light, erythema, low energy, sensitive skin

## Abstract

**Background:**

Sensitive skin (SS) is a multifactorial syndrome that affects about half of the world's population. However, there is no standardized treatment protocol. Photovoltaic technology has been widely used in recent years for the treatment of sensitive skin, but the efficacy of low‐energy delicate pulsed light (DPL) in the treatment of sensitive skin is unknown.

**Objective:**

To explore the efficacy and safety of low‐energy DPL treatment for sensitive skin.

**Methods:**

This study included 181 patients with SS who attended the Department of Dermatology from January 2019 to January 2022. All patients were treated with DPL (5.0–6.8 J/cm^2^). Patients received varying durations of DPL, with 4‐week intervals based on the severity of their condition. Sensitivity scores at baseline and final treatment were collected using a 10‐item sensitivity scale (SS‐10). Facial erythema was assessed through the Clinician Erythema Assessment Scale (CEA) based on facial photographs taken at each visit. VISIA‐CR was used to visualize and analyze facial redness at baseline and after the final treatment, and erythema values were recorded. Adverse reactions were documented during each treatment session.

**Results:**

Among 181 patients, 86 were SS alone, 32 were rosacea with SS, 32 were acne with SS, and 31 were dermatitis with SS. A significant difference was observed between patients' baseline and final SS‐10 scores (*p* < 0.001). Statistically significant differences were found between the patients' CEA scores at different visits (*p* < 0.001). Clinical photographs demonstrated significant improvements in facial redness and skin color. VISIA‐CR analysis showed a considerable reduction in erythema values at the final treatment visit compared to baseline (*p* < 0.001). No obvious adverse effects were observed in any of the patients.

**Conclusion:**

Low‐energy DPL is an effective and safe treatment for sensitive skin.

## 1 Introduction

Sensitive skin (SS) is a condition characterized by heightened intolerance to external stimuli, resulting in unpleasant sensations such as stinging, burning, pain, and itching. The skin may appear normal or present with erythema, primarily affecting the face [1]. SS can be classified as primary or secondary, with approximately 66% of women with atopic dermatitis, 57% with rosacea, and 31.8% with acne experiencing it [2]. Epidemiological studies conducted in the United Kingdom, the United States, and France report prevalence rates of SS as approximately 40% in men and 60% in women [3]. A meta‐analysis by Brenaut et al. [4] identified multiple triggers for SS, including physical, chemical, and psychological factors, with cosmetics being the most significant, followed by humid air. The exact mechanisms of SS remain unclear; however, current research suggests that it involves a complex process of skin barrier dysfunction, neurovascular responses, and innate immune inflammation [3]. Without intervention, SS can persist for years and have significant psychological, social, and economic effects. Because of the multifactorial pathogenesis of SS, there is currently no standardized treatment protocol. Treatments include avoidance of exacerbating factors, skin barrier repair, photoprotection, and anti‐inflammatory agents; however, pharmacological treatments have a slow onset of action, can lead to adverse effects with long‐term use, and demonstrate limited efficacy in managing facial erythema. Therefore, safer and more effective treatment options are required.

In recent years, phototherapy has been introduced as a potential treatment for SS. Unlike traditional photothermal therapies, which rely on heat‐induced tissue damage and subsequent repair, photomodulation utilizes nonthermal mechanisms. This approach employs specific pulse modes, pulse widths, and low‐energy narrow‐spectrum light sources to regulate cellular activity, thereby achieving therapeutic effects. Intense pulsed light (IPL) is a noncoherent polychromatic broadband filtered flash lamp that emits light in the wavelength range of approximately 400–1200 nm [5]. IPL can be absorbed by water, hemoglobin, and melanin in the skin tissue and is widely used to treat pigmentary and vascular lesions. Delicate pulsed light (DPL) refines the IPL by filtering out excess spectra, allowing only the 500–600 nm wavelength band to pass through. This improvement offers more precise targeting of lesions with a reduced risk of thermal damage, higher safety, and greater energy efficiency. DPL has been proven to be effective and safe in treating facial telangiectasia. However, research on low‐energy DPL in SS is limited. Consequently, this study aimed to evaluate the efficacy and safety of low‐energy DPL photomodulation in treating SS.

## 2 Materials and Methods

### 2.1 General Information

Patients with SS treated at the Department of Dermatology, from January 2019 to January 2022, were selected for retrospective analysis. These patients exhibited Fitzpatrick skin types III or IV. This study was approved by the Ethics Committee, and all procedures were performed in accordance with the Declaration of Helsinki.

### 2.2 Inclusion and Exclusion Criteria

Inclusion criteria included the following: diagnosis of SS; no prior facial light or laser therapy; No other treatments or medications administered during the observation period.

Exclusion criteria included the following: skin infection in the treatment area; use of photosensitive or retinoid drugs within the past 3 months; pregnant or lactating women; history of photosensitivity or severe systemic diseases; active skin diseases, such as connective tissue disorders or autoimmune diseases; malignant skin conditions; psychological disorders; contraindications to various types of lasers.

### 2.3 Equipment and Treatment Protocol

Treatment was performed using a Brilliant Laser Photonics Workstation (Alma Lasers, IL), with the Delicate Skin System set to a wavelength of 500–600 nm. The spot size was 6 mm, pulse width 10–15 ns, cooling set at 50%, and energy density ranged from 5.0 to 6.8 J/cm^2^. Before treatment, the patients were informed of potential adverse reactions and posttreatment care, and they signed an informed consent form. During the procedure, facial skin was cleansed; then, a uniform layer of cooling gel was applied to the face. Treatment parameters, including energy levels, were customized based on the patient's lesion characteristics, skin color, Fitzpatrick skin types, and individual response. The parameters were adjusted to ensure that patients experienced no symptoms or only mild warmth. The endpoint of treatment was defined as either no visible reaction or mild redness. After treatment, ice packs were applied for 20 min to alleviate skin redness. For 1 week following treatment, patients were instructed to use a soothing facial mask nightly for 15 min. They were also advised to avoid skin care and cosmetic products with irritating ingredients, strictly adhere to sun protection measures, and maintain hydration and moisturization. Daily cleansing with water and the use of barrier repair creams are recommended. Treatment was administered once a month, with four sessions constituting one treatment course. The number of treatments varied depending on the patient's skin condition.

### 2.4 Sensitivity and Efficacy Assessment

The 10‐item Sensitive Scale (SS‐10) was used to assess sensory symptoms over the past 3 days through visual analog scoring. These symptoms included visible signs (redness) and subjective symptoms (skin irritability, stinging, burning, tautness, itching, pain, general discomfort, and flashes) [6]. Each item is scored from 0 to 10, where 0 indicates “none” and 10 indicates “maximum irritation.” An SS‐10 score greater than 13 can be used as the cutoff to diagnose SS, and a score greater than 5 indicates mild sensitivity [7]. The SS‐10 scores were collected at baseline and after the final treatment, either in‐person or remotely. Treatment effectiveness was calculated using the efficacy index (EI). The formula used is as follows:

$$\begin{array}{c} \mathrm{EI}=\left(\text{total score before treatment}-\text{total score after treatment}\right)\\ /\text{total score before treatment}\times 100\%. \end{array}$$

The EI scores indicated the following: cure: EI ≥ 90%; obvious effectiveness: 60% ≤ EI < 90%; improvement: 30% ≤ EI < 60%; ineffective: EI < 30%. The overall efficacy rate was calculated as follows:

$$\begin{array}{c} \left(\text{Number of cures}+\text{obvious effectiveness}+\text{improvement}\right)\\ /\text{Total number of patients}. \end{array}$$

### 2.5 Erythema Grading Assessment and Erythema Value

Clinical photographs were captured at each time point using identical camera settings, lighting, and patient positioning. For each treatment session, two blinded physicians who did not participate in the treatment evaluated the photographs according to the following standards and compared them to those at the baseline visit. The Clinician Erythema Assessment Scale (CEA) was used to objectively assess the improvement in skin erythema with DPL [8]: 0 = clear skin with no signs of erythema; 1 = almost clear with slight redness; 2 = mild erythema with definite redness; 3 = moderate erythema with marked redness; and 4 = severe erythema and fiery redness. Erythema values were recorded through VISIA‐CR (Canfield Scientific, USA) to photograph and analyze the red areas of the skin at baseline and after the final treatment.

### 2.6 Safety Assessment

Each visit involved a visual inspection of the treatment area. Adverse reactions related to the treatment, including skin pain, burning, redness, swelling, blistering, and scarring, were recorded.

### 2.7 Statistical Analysis

Data were analyzed using SPSS version 26.0 (IBM, USA). The paired‐sample *t*‐test was used to evaluate the SS‐10 scores and facial erythema values. Repeated‐measures ANOVA was used to evaluate CEA scores. The results were presented as mean ± SD. Statistical significance was set at *p* < 0.05.

## 3 Results

### 3.1 General Information

Among 181 SS patients, 24 were men and 157 were women, with ages ranging from 16 to 53 years and an average age of 31.42 ± 8.18 years. All lesions were located on the face. One hundred twenty‐six patients had Fitzpatrick skin type III (70%), and 55 patients had Fitzpatrick skin type IV (30%). There were 86 patients with SS alone, 32 with rosacea and SS, 32 with acne and SS, and 31 with unspecified dermatitis and SS. Distributions of sex and age are shown in Table 1.

**TABLE 1:** Patient characteristics.

Patient	Men	Women	Total	Age (mean ± SD)
SS alone	8	78	86	31.73 ± 7.01
Rosacea with SS	5	27	32	34.50 ± 10.53
Acne with SS	7	25	32	25.63 ± 5.62
Dermatitis with SS	4	27	31	33.35 ± 8.05
Total	24	157	181	31.42 ± 8.18

### 3.2 SS‐10

**FIGURE 1:**
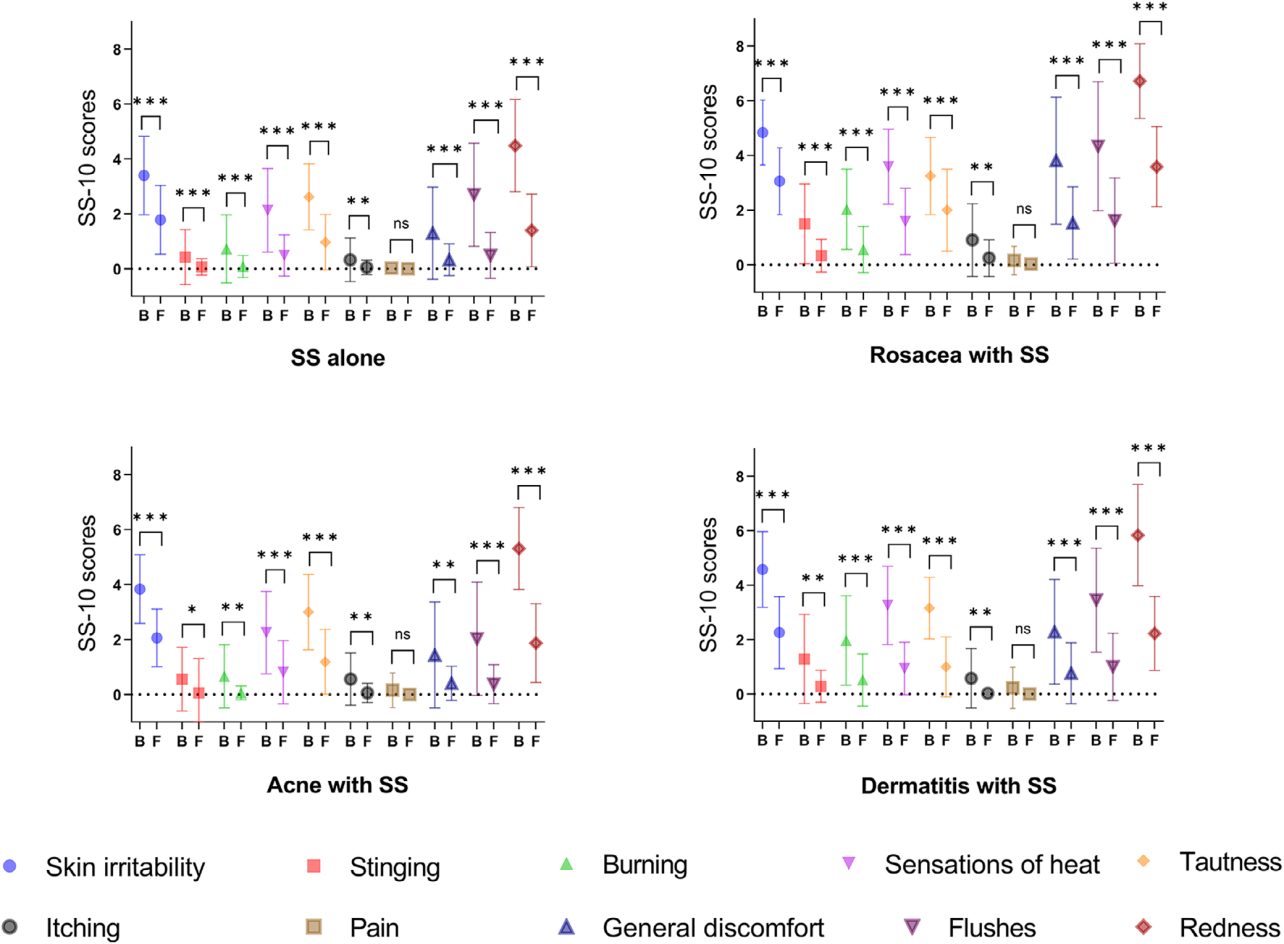
Improvement in all symptoms and signs of the SS‐10 after the patient's final treatment. The *y*‐axis represents the patients' scores for each index, expressed as mean ± SD. A paired‐sample *t*‐test was used to compare baseline (B) and final visits (F) to obtain a *p*‐value (ns, *p* > 0.05; *, *p* < 0.05; **, *p* < 0.01; ***, *p* < 0.001).

**FIGURE 2:**
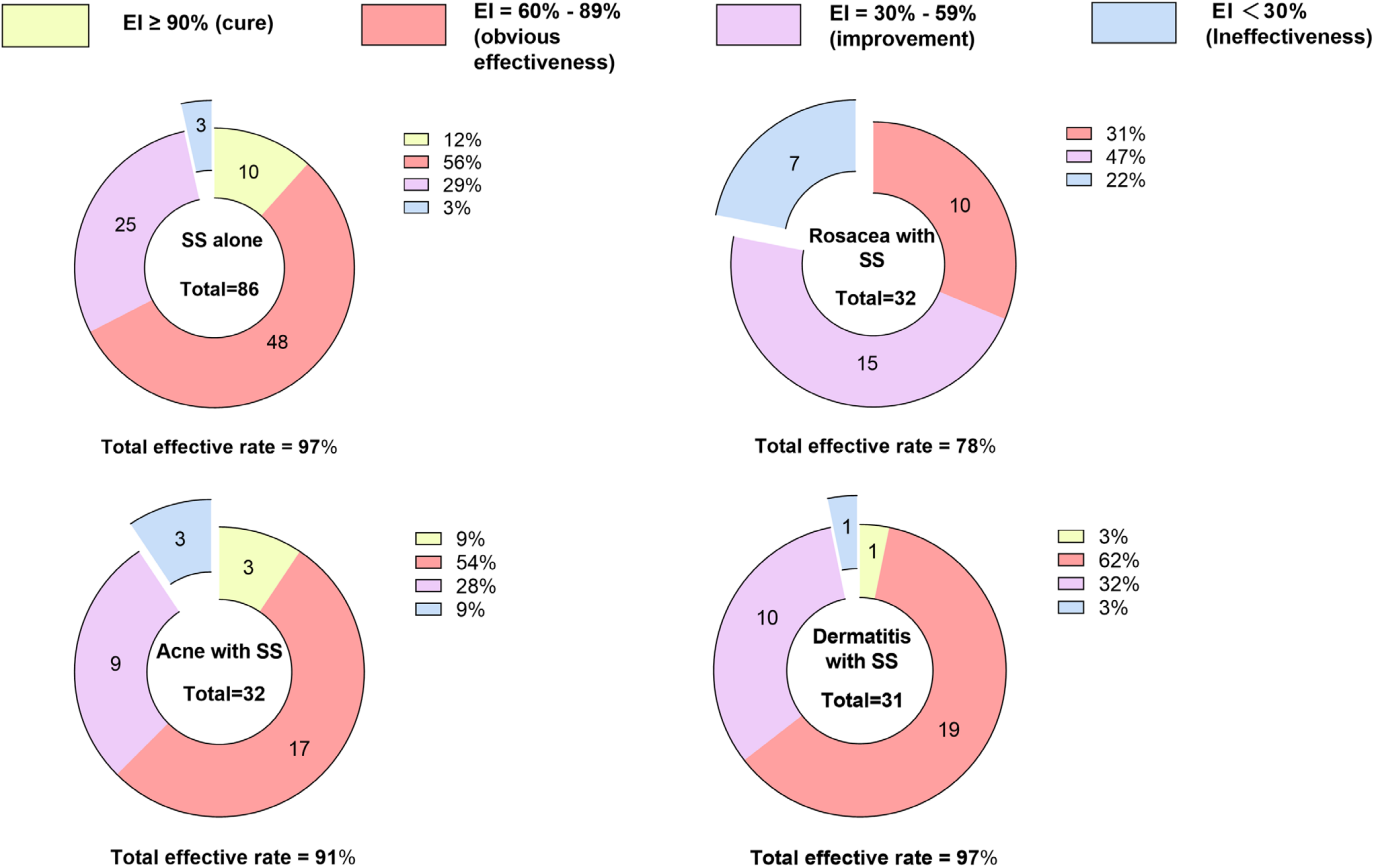
Efficacy classification statistics after treatment. Efficacy index (EI) = (total score before treatment − total score after treatment)/total score before treatment × 100%; Total effective rate = (number of cures + obvious effectiveness + improvement)/total number of patients.

DPL was administered at varying durations according to the severity of each patient's condition and their recovery after treatment. One hundred eighty‐one SS patients received a maximum of four treatments and a minimum of two. There were statistical differences between baseline treatment sensitivity scores and the final posttreatment sensitivity scores of patients with SS alone (18.15 ± 8.40 vs. 5.66 ± 4.40, *t* = 15.29, *p* < 0.001), patients with rosacea with SS (31.16 ± 9.07 vs. 14.59 ± 7.41, *t* = 9.38, *p* < 0.001), patients with acne with SS (19.81 ± 8.95 vs. 6.91 ± 4.44, *t* = 8.53, *p* < 0.001), and patients with dermatitis with SS (26.65 ± 9.44 vs. 9.03 ± 6.35, *t* = 12.93, *p* < 0.001) (Table 2). After the final treatment, all the patients' scores of redness, irritation, tingling, burning, tautness, itching, general discomfort, and hot flashes significantly decreased (*p* < 0.05), as shown in Figure 1. No significant difference was found in pain scores before and after treatment (*p* > 0.05). An EI greater than 30% was observed in 83 patients with SS alone, 25 patients with rosacea with SS, 29 patients with acne with SS, and 30 patients with dermatitis with SS. The total efficacy rates of patients with SS alone, patients with rosacea with SS, patients with acne with SS, and patients with dermatitis with SS were 97%, 78%, 91%, and 97%, respectively. Detailed efficacy classifications are presented in Figure 2.

**TABLE 2:** Comparison of SS‐10 total scores of patients between baseline and final visit (mean ± SD).

Patient	Sensitivity score		Baseline visit versus final visit			
	Baseline visit	Final visit	Mean difference (95% CI^a^)	*t*	*p* ^b^	*d* ^c^
SS alone	18.15 ± 8.40	5.66 ± 4.40	1.28 (10.87, 14.11)	15.29	< 0.001	1.65
Rosacea with SS	31.16 ± 9.07	14.59 ± 7.41	16.56 (12.96, 20.17)	9.38	< 0.001	1.66
Acne with SS	19.81 ± 8.95	6.91 ± 4.44	8.56 (9.82, 16.00)	8.53	< 0.001	1.51
Dermatitis with SS	26.65 ± 9.44	9.03 ± 6.35	17.61 (14.83, 20.40)	12.93	< 0.001	2.32

^a^ 95% CI indicates 95% confidence interval.

^b^ A paired‐sample *t*‐test was used to compare baseline and final visits to obtain a *p*‐value.

^c^ *d* represents Cohen's *d*.

### 3.3 CEA and Erythema Values

**FIGURE 3:**
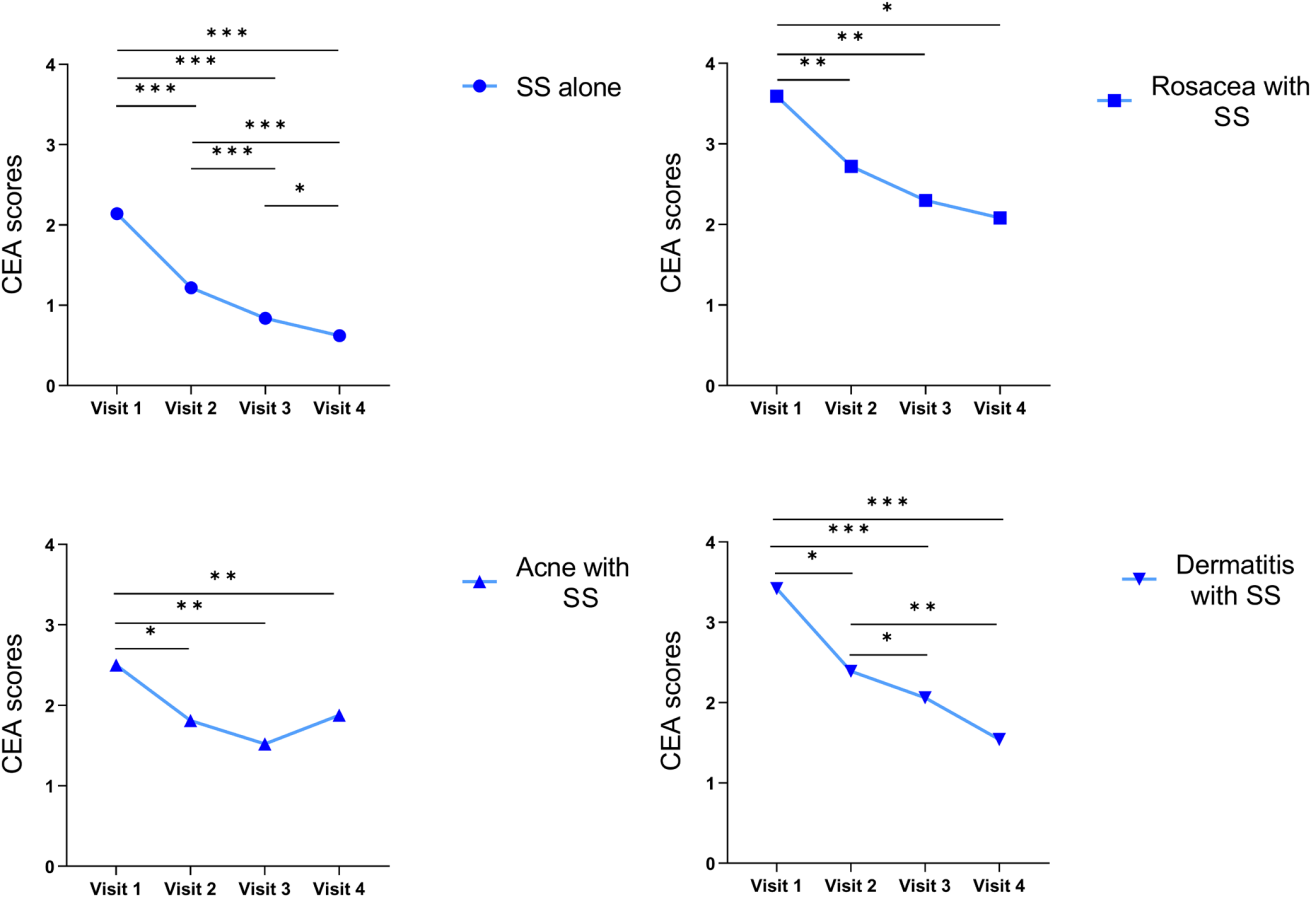
Comparison of CEA scores between adjacent visits in SS patients. Facial erythema improved in all patients after initial treatment, and CEA scores for facial erythema decreased progressively at the subsequent visit in most patients. *p*‐Values were calculated using repeated measures ANOVA for two‐by‐two comparisons between visits (*, *p* < 0.05; **, *p* < 0.01; ***, *p* < 0.001).

**FIGURE 4:**
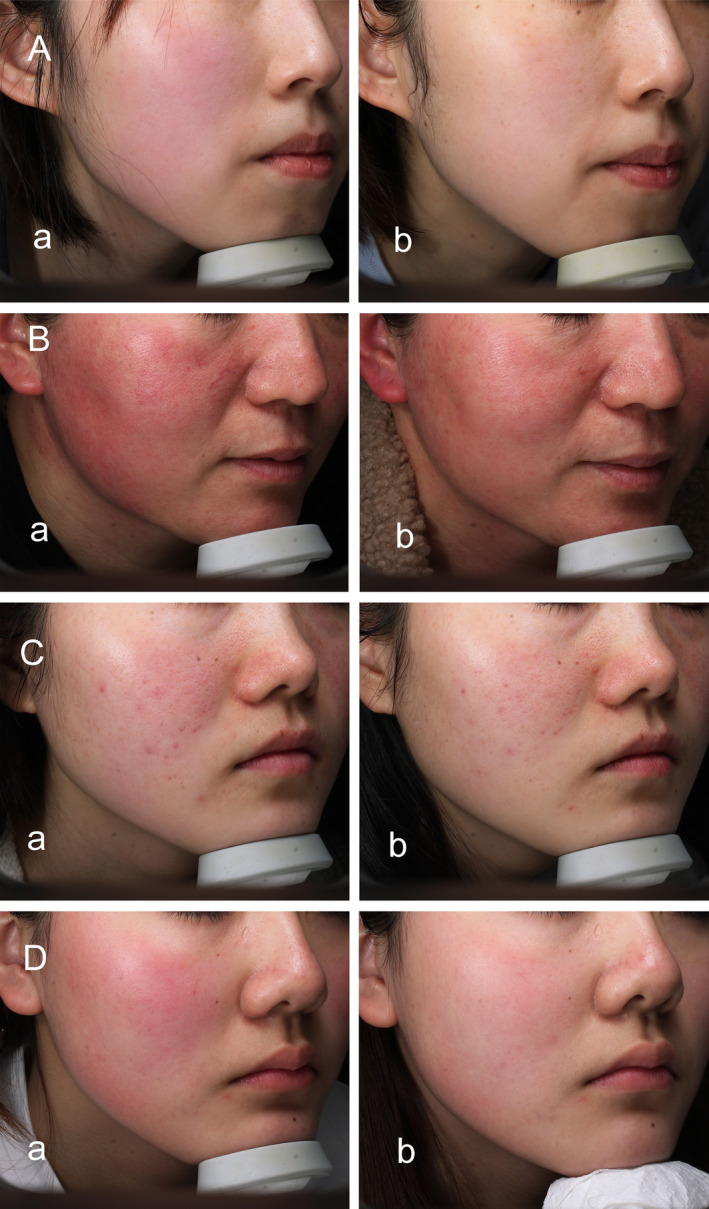
Facial erythema improved significantly after treatment. (A) SS alone; (B) rosacea with SS; (C) acne with SS; (D) dermatitis with SS. Photographs of baseline (Aa, Ba, Ca, Da) and final (Ab, Bb, Cb, Db) treatments showed significant improvement in facial erythema.

**FIGURE 5:**
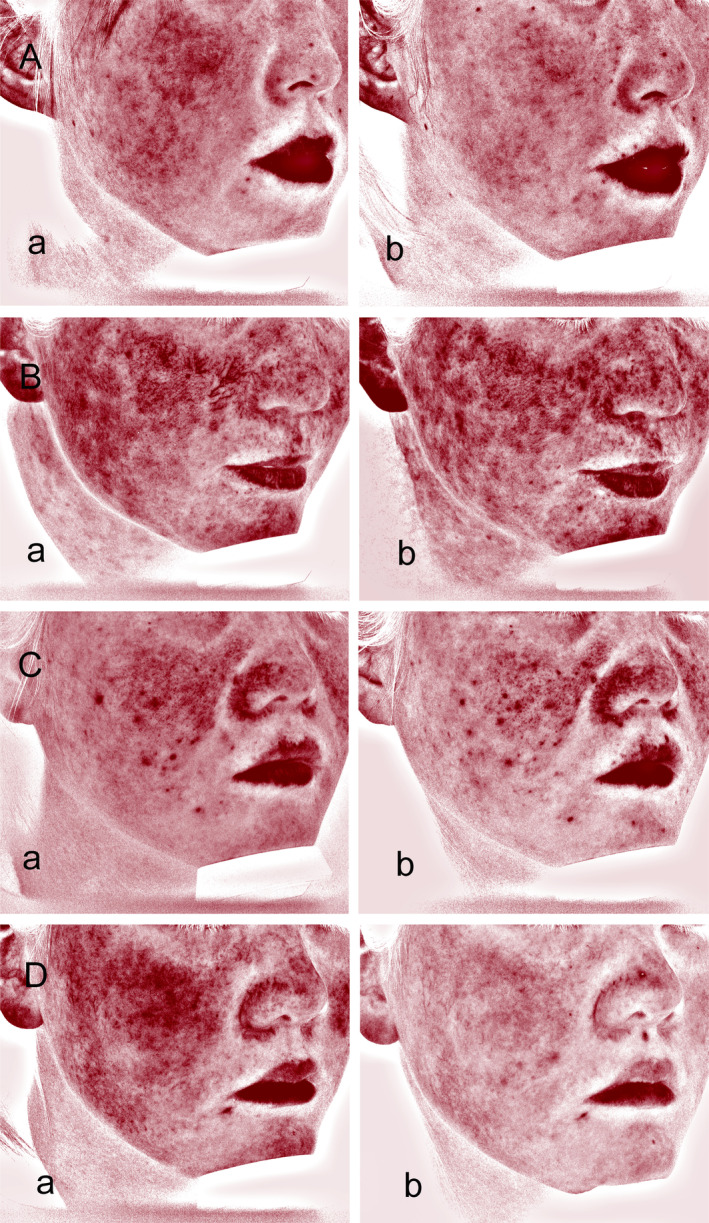
VISIA‐CR analysis of facial images before and after treatment. (A) SS alone; (B) rosacea with SS; (C) acne with SS; (D) dermatitis with SS. Reduced erythema area and color reduction at final treatment (Ab, Bb, Cb, Db) compared to baseline treatment (Aa, Ba, Ca, Da).

Based on the erythema grading assessments at each visit, most patients showed an improvement in facial redness. During the four patient visits, statistical differences in CEA scores were found in all four groups of patients (*p* < 0.001, Table 3). Owing to variation in patient numbers between visits, an analysis was conducted comparing adjacent visits (Figure 3). Statistically significant differences were found between baseline and final CEA treatment scores in all patient groups (*p* < 0.001). The erythema grading assessments of patients with SS alone showed significant differences between Visit 1 and Visits 2, 3, and 4 (*p* < 0.001); between Visit 2 and Visits 3 and 4 (*p* < 0.001); and between Visit 3 and Visit 4 (*p* = 0.039). Also, the erythema grading assessments of patients with rosacea with SS or acne with SS all have significant differences between Visit 1 and Visits 2, 3, and 4 (*p* < 0.05). However, no significant difference in CEA scores was found between Visit 2 and Visits 3 and 4 (*p* > 0.05) or between Visit 3 and Visit 4 (*p* > 0.05). For patients with dermatitis and SS, significant differences were identified between Visit 1 and Visits 2, 3, and 4 (*p* < 0.05) and between Visit 2 and Visits 3 and 4 (*p* < 0.05), whereas no significant difference was observed between Visit 3 and Visit 4 (*p >* 0.05). As shown in Figure 4, facial redness was significantly alleviated after the final treatment of patients with SS alone and those with SS with other diseases. The VISIA‐CR analysis of facial erythema value at baseline and after final treatment showed statistical differences in the front, left, and right views (*p* < 0.001), as presented in Table 4. VISIA‐CR images indicated deep red spots and patches that lightened after treatment (Figure 5).

**TABLE 3:** Comparison of CEA scores between visits in SS patients (mean ± SD).

Patient	Visit 1	Visit 2	Visit 3	Visit 4	*F*	*p* ^a^	Partial *η* ^2^ ^b^
SS alone	2.14 ± 1.03	1.22 ± 1.02	0.84 ± 0.77	0.62 ± 0.75	85.79	< 0.001	0.69
Rosacea with SS	3.59 ± 0.56	2.72 ± 0.81	2.30 ± 1.13	2.08 ± 1.44	9.16	< 0.001	0.45
Acne with SS	2.50 ± 1.05	1.81 ± 1.06	1.52 ± 1.03	1.88 ± 0.64	8.83	< 0.001	0.56
Dermatitis with SS	3.42 ± 0.89	2.39 ± 1.09	2.06 ± 1.00	1.54 ± 0.97	29.6	< 0.001	0.71

^a^ *p*‐Values were calculated using repeated‐measures ANOVA between visits.

^b^ *Partial η* ^2^ represented partial eta squared.

**TABLE 4:** Comparison of facial erythema values between baseline and final visit.

Patient	View	Baseline visit (*N* = 181)	Final visit (*N* = 181)	Baseline visit versus final visit			
				Mean difference (95% CI)	*t*	*p* ^a^	*d*
SS alone	Front view	41.92 ± 10.49	34.33 ± 10.14	7.59 (6.27, 8.91)	11.47	< 0.001	1.24
	Left view	46.97 ± 7.17	39.19 ± 6.82	7.78 (6.70, 8.85)	14.39	< 0.001	1.55
	Right view	46.27 ± 7.08	37.84 ± 6.61	8.43 (7.51, 9.35)	18.27	< 0.001	1.97
Rosacea with SS	Front view	52.43 ± 8.60	42.66 ± 10.46	9.77 (6.97, 12.57)	7.13	< 0.001	1.26
	Left view	53.93 ± 8.03	46.39 ± 7.05	7.54 (5.64, 9.45)	8.09	< 0.001	1.43
	Right view	54.50 ± 7.37	45.60 ± 7.13	8.9 (6.76, 11.03)	8.50	< 0.001	1.50
Acne with SS	Front view	41.27 ± 8.69	34.65 ± 7.36	6.62 (4.75, 8.49)	7.22	< 0.001	1.28
	Left view	46.69 ± 7.72	38.79 ± 6.86	7.9 (6.10, 9.70)	8.95	< 0.001	1.58
	Right view	47.98 ± 7.68	39.83 ± 6.78	8.15 (6.72, 9.57)	11.69	< 0.001	2.07
Dermatitis with SS	Front view	47.59 ± 10.85	41.63 ± 10.52	5.96 (4.35, 7.57)	7.57	< 0.001	1.36
	Left view	54.35 ± 9.91	47.09 ± 9.51	7.27 (5.20, 9.33)	7.19	< 0.001	1.29
	Right view	51.96 ± 8.74	42.24 ± 7.69	9.72 (7.61, 11.83)	9.40	< 0.001	1.69

*Note:* Values are presented as mean ± SD.

^a^ A paired‐sample *t*‐test was used to compare baseline and final visits to obtain a *p*‐value.

### 3.4 Adverse Reactions

Most patients experienced immediate facial redness following treatment, which significantly improved after 20 min of ice application. Some patients developed facial swelling that resolved within 3 days. No serious adverse reactions, such as blisters, abnormal pigmentation, or scarring, were reported.

## 4 Discussion

SS is a common multifactorial syndrome that occurs under various physiological and pathological conditions. The likelihood of SS in acne patients is 2.5 times higher than in controls [9], and the prevalence of SS among rosacea patients can reach 100% [10]. Approximately 71% of adults report having SS, with approximately 40% experiencing moderate to severe sensitivity [11]. The high prevalence of SS highlights its importance as a public health concern. Current clinical treatments for SS mainly include health education, proper skin care, pharmacotherapy, and physical treatments, with no established gold standard. With the exploration of photoelectric technology for the treatment of skin diseases, photomodulation has recently been applied to SS. This study utilized low‐energy DPL to treat patients with SS and found it to be effective in improving facial sensitivity and redness, with no significant adverse reactions.

The pathophysiological mechanisms of SS are complex and include impaired skin barrier function, increased neurovascular reactivity, immune‐inflammatory responses, and microecological disorder [3, 12]. Impaired skin barrier function is one of the primary causes of SS. The skin barrier is the first line of defense against external stimuli, and its impairment not only increases the permeability to allergens or irritants but also accelerates transepidermal water loss. Photomodulation involves narrow‐band light with specific pulse sequences and durations, which provide a nonthermal stimulus to induce skin rejuvenation effects. It works by using an appropriate “package” of photons to stimulate mitochondrial organelles, which upregulates or downregulates mitochondrial gene activity, thereby altering cellular activity [13]. Photomodulation has been shown to have a significant effect on promoting collagen synthesis, wound healing, sealing blood vessels, repairing skin barrier function, and exerting anti‐inflammatory properties.

IPL treatment penetrates the epidermis and is absorbed by hemoglobin [14], thereby reducing capillary dilation without damaging other skin tissues [15]. Its thermal effect increases the synthesis of type I and type III collagen in the dermis [15], induces the expression of various heat shock proteins [16], and reduces the release of inflammatory mediators [17]. Therefore, IPL therapy is considered the optimal choice for treating SS. However, IPL has several limitations, including its broad spectral ranges, nonuniform energy distribution, higher risks of side effects, and the requirement for more treatment sessions. Compared to broad‐spectrum IPL, DPL has a wavelength range of 500–600 nm, allowing for more precise targeting of lesions, enhanced safety, improved clinical efficacy, and reduced patient discomfort. Most patients tolerate DPL well, and fewer sessions are typically required. Tsunoda et al. [18] reported three cases of diffuse erythema treated with narrow‐band pulsed light (500–635 nm), which showed good results in reducing erythema without damaging the surrounding tissues. In contrast, Thaysen‐Petersen et al. found that the risk of erythema and pain intensity increased with the energy density of the pulsed light, particularly in patients with darker skin tones [19]. Patients with SS have an overall reduced heat pain threshold compared to those with healthy skin and are more sensitive to various stimuli [20]. To avoid adverse events and improve patient compliance, gentle low‐energy DPL was used.

The symptoms of SS are primarily subjective and transient, making patient self‐reporting the most effective method for evaluation. The SS‐10 score allows for timely adjustments to the treatment plan. Our study demonstrated that after low‐energy DPL treatment, facial symptoms such as tingling, burning, and tightness significantly improved (*p* < 0.05). This is attributed to the fact that DPL may activate energy mechanisms during mitosis, provide more energy for cellular activities, enhance fibroblast metabolic activity, upregulate fibroblast collagen production, and regulate matrix metalloproteinase activity and inflammatory factor expression, thus promoting skin healing and improving impaired skin barriers. A clinical study in Korea involving 30 patients with SS found that low‐intensity light therapy significantly improved symptoms such as facial tingling, burning, and redness, with almost complete resolution of cosmetic intolerance [21]. Feng et al. found that IPL treatment increased type I and type III collagen levels, and reduced elastin content but improved elastin fiber arrangement. Transmission electron microscopy showed increased fibroblast activity, with a greater number of collagen fibers neatly rearranged within the stroma [22]. These results were replicated by Faucz et al. [23] One study found that IPL, when applied directly to fibroblasts cultured in vitro, significantly promotes cell proliferation as well as collagen and hyaluronic acid secretion [24]. Hyaluronic acid binds to a large number of water molecules and plays a significant role in skin hydration. Patients with SS exhibit impaired skin barrier function and increased transepidermal water loss, and hyaluronic acid may help improve this condition.

In our study, facial erythema in patients with SS significantly improved after the first treatment (*p* < 0.05), with redness gradually diminishing with an increasing number of treatment sessions. This is likely because the DPL wavelength of 500–600 nm corresponds to the absorption peak of hemoglobin. Additionally, DPL can convert ineffective spectral energy at the ends into effective therapeutic spectra, resulting in higher energy targeting superficial erythema and vascular dilation. This leads to the coagulation of hemoglobin and closure of dilated capillaries, thus effectively removing superficial erythema and capillary dilation. Choi et al. found that low‐intensity light could inhibit keratinocyte secretion of vascular endothelial growth factor (VEGF) [21]—a multifunctional cytokine upregulated in the epidermis of inflammatory skin diseases such as psoriasis, rosacea, contact dermatitis, and atopic dermatitis [25]. VEGF can increase vascular permeability and may be associated with persistent erythema. Furthermore, IPL treatment can regulate the skin microbiome, reduce the abundance of *Propionibacterium acnes* , and increase *Staphylococcus epidermidis* population [26]. IPL treatment improves inflammation and increases transforming growth factor beta (TGF‐β) expression [27]. TGF‐β plays a crucial role in suppressing inflammatory responses, regulating immune responses, and participating in wound healing and tissue repair [25]. Several studies have confirmed the effectiveness of IPL therapy in treating acne and rosacea. Kim, Lee, and Choi [28] found that IPL not only prevents excessive pigmentation but also reduces excessive skin immune responses by suppressing the expression of proinflammatory cytokines (IL‐6, IL‐7, and IL‐8). Additionally, the use of soothing face masks after treatment helps to moisturize and repair the skin barrier.

Although DPL treatment is critical for improving facial sensitivity and erythema in patients with SS, adjunctive skin care measures, including gentle cleansing, moisturization, and photoprotection, are equally important [29]. In this study, while DPL treatment was performed, patients were provided with health education and skin care guidance. It has been reported that SS can be influenced by environmental factors such as dust, air pollutants, dryness, and high temperatures [30]. Proper cleansing helps maintain facial hygiene. A study demonstrated that mild cleansers and moisturizers can effectively alleviate symptoms such as dryness, stinging, tightness, and erythema in patients with SS while enhancing skin hydration and barrier repair [31]. Consequently, all patients routinely applied soothing masks and barrier‐repair creams after DPL treatment to expedite the recovery skin barrier. Furthermore, ultraviolet (UV) radiation can impair skin barrier function and patients with SS tend to exhibit reduced tolerance to UV exposure. So it is advised to apply strict photoprotection measures after DPL treatment, including gentle, non‐irritating sunscreens or protective clothing, to mitigate UV‐related damage.

This study had certain limitations. First, the number of male patients was relatively small. This may be attributed to the thicker epidermis in men, making SS less common [32]. As a result, fewer men seek medical attention for SS. Additionally, cosmetics, a major trigger for SS, are predominantly used by women, resulting in a higher prevalence of SS among women [11]. Second, this study was a retrospective analysis, and some patients' sensitivity symptoms and facial erythema may have improved over time. Third, the patients were recruited from a single hospital, limiting generalizability. Multicenter studies with larger sample sizes and longer follow‐up durations are needed to obtain more objective and reliable results. Further prospective studies with control groups would enhance the validity and reliability of these results. Despite these limitations, this study is the first to demonstrate the effectiveness of low‐energy DPL in improving sensitivity and facial erythema in patients with SS, providing a safe and effective method for clinical SS treatment and theoretical support for low‐energy DPL therapy.

## 5 Conclusion

1. SS is a syndrome characterized by subjective and objective symptoms that occur when the skin is exposed to weak external stimuli. It is prevalent on the face, and its incidence is increasing annually.
2. Low‐energy DPL can effectively improve the skin sensitivity of patients with SS.
3. Low‐energy DPL can effectively improve the facial erythema of these patients without causing obvious adverse reactions.
4. It provides an effective and safe treatment solution for sensitive skin.

## Author Contributions

**Yujie Fu:** writing – original draft, validation, formal analysis, visualization, data curation, methodology. **Yue Quan:** writing – original draft, validation, formal analysis, visualization, data curation. **Wanxing Zhao:** investigation, formal analysis, validation, software. **Zhaoyang Liu:** methodology, validation, software. **Jiao Peng:** investigation, validation, software. **Xinyue Pang:** investigation, validation. **Boyu Zhao:** investigation, software. **Lisha Tan:** investigation, validation. **Quan Zhou:** resources, supervision. **Lili Shao:** resources, supervision. **Huiping Wang:** supervision, funding acquisition. **Shuping Hou:** methodology, writing – review and editing, formal analysis, funding acquisition, resources, supervision, project administration.

## Ethics Statement

The study was performed in accordance with the ethical standards stipulated in the 1964 Declaration of Helsinki and received approval from the Medical Ethics and Human Research Committee of Tianjin Medical University General Hospital of China (IRB2024‐YX‐358‐01). All patients provided written informed consent.

## Conflicts of Interest

The authors declare no conflicts of interest.

## Data Availability

The data that support the findings of this study are available from the corresponding author upon reasonable request. The data are not publicly available due to privacy or ethical restrictions.
